# Practical Tools to Implement Massive Parallel Pyrosequencing of PCR Products in Next Generation Molecular Diagnostics

**DOI:** 10.1371/journal.pone.0025531

**Published:** 2011-09-30

**Authors:** Kim De Leeneer, Joachim De Schrijver, Lieven Clement, Machteld Baetens, Steve Lefever, Sarah De Keulenaer, Wim Van Criekinge, Dieter Deforce, Filip Van Nieuwerburgh, Sofie Bekaert, Filip Pattyn, Bram De Wilde, Paul Coucke, Jo Vandesompele, Kathleen Claes, Jan Hellemans

**Affiliations:** 1 Center for Medical Genetics, Ghent University, Ghent, Belgium; 2 NXTGNT, Ghent University, Ghent, Belgium; 3 Biobix, Laboratory for Bioinformatics and Computational Genomics, Ghent University, Ghent, Belgium; 4 Biostat, Department of Applied Mathematics, Biometrics and Process Control, Ghent University, Ghent, Belgium; 5 Laboratory for Pharmaceutical Biotechnology, Ghent University, Ghent, Belgium; Massachusetts General Hospital, United States of America

## Abstract

Despite improvements in terms of sequence quality and price per basepair, Sanger sequencing remains restricted to screening of individual disease genes. The development of massively parallel sequencing (MPS) technologies heralded an era in which molecular diagnostics for multigenic disorders becomes reality. Here, we outline different PCR amplification based strategies for the screening of a multitude of genes in a patient cohort. We performed a thorough evaluation in terms of set-up, coverage and sequencing variants on the data of 10 GS-FLX experiments (over 200 patients). Crucially, we determined the actual coverage that is required for reliable diagnostic results using MPS, and provide a tool to calculate the number of patients that can be screened in a single run. Finally, we provide an overview of factors contributing to false negative or false positive mutation calls and suggest ways to maximize sensitivity and specificity, both important in a routine setting. By describing practical strategies for screening of multigenic disorders in a multitude of samples and providing answers to questions about minimum required coverage, the number of patients that can be screened in a single run and the factors that may affect sensitivity and specificity we hope to facilitate the implementation of MPS technology in molecular diagnostics.

## Introduction

A multitude of laboratory technologies for the detection of DNA mutations have been developed over the last decades. In current diagnostic settings, most frequently a combination of a mutation scanning technique, followed by Sanger sequencing of the abnormal DNA fragments is used. Well known examples of widely used methods to identify the aberrant fragments are single strand conformation polymorphism (SSCP), conformation sensitive gel electrophoresis (CSGE), high performance liquid chromatography (HPLC) and more recently high resolution melting curve analysis (HRMCA) [1], [2], [3], [4]. Despite its higher cost, Sanger sequencing [5] of DNA fragments remains the preferred method for mutation analysis because of its superior sensitivity and specificity and the detailed sequence information that can be obtained in a single step approach. Improvements on sequencing chemistries, instruments and data analysis software, as well as increases in throughput and reductions in cost resulted in the adoption of this technology for routine mutation analysis for monogenic diseases. However, expansion of molecular diagnostics to the realm of multigenic disorders requires the implementation of new methods with increased mutation detection efficiency but without a decrease in cost efficiency. Massively parallel sequencing (MPS) technologies (see [6], [7] for an overview) are an interesting alternative because of their higher throughput and lower cost per base as compared to Sanger sequencing. In addition, throughput and cost for MPS technologies per base are rapidly evolving (from 0.1 Gb per run for the Roche Genome Sequencer at the end of 2006 to 150–300 Gb per run for Illumina's HiSeq2000 and ABI's 5500XL platform in 2011) at a speed vastly surpassing the evolution rate seen in semiconductor industries (Moore's law).

In order for MPS to take over the role of Sanger sequencing and to evolve into the method of choice for next generation molecular diagnostics (NGMD), a number of hurdles need to be taken and questions be answered. The goal of this paper is to remove a number of these obstructions by describing strategies which enable mutation analysis through MPS, by presenting tools for determination of the required coverage and the number of patients who can be screened in a single run, and by listing possible sources of false negative or false positive mutation calls along with possible solutions. The guidelines and tools provided in this study were formulated or calculated based on pyrosequencing data obtained on the GS-FLX instrument (454-Roche), but may provide better insights into applications with other MPS chemistries as well.

## Materials and Methods

### Generation of sequencing data

#### Sample preparation

The data presented in this article are derived from 10 GS-FLX sequencing runs (using both Standard and Titanium chemistries) on samples prepared with different approaches. In total over 200 patient samples were evaluated in these 10 experiments. To pool different patients in a single experiment multiplex identifier (MID)-tags were attached on all patients' samples. Different approaches were evaluated to attach these tags:

Approach 1: the samples investigated for recessive congenital deafness (15 genes: *GJB2*, *SLC26A4*, *MYO15A*, *OTOF*, *CDH23*, *TMC1*, *TMPRSS3*, *TECTA*, *TRIOBP*, *TMIE*, *PJVK*, *ESPN*, *PCDH15*, *ESRRB*, *MYO7A* - 643 amplicons) were prepared with PCR (Kapa Taq kit (Sopachem)) followed by an adapter ligation approach. All PCR products for a given sample are pooled, thereby reducing the number of parallel reactions in the next step from the number of sample-amplicon combination (SAC) to the number of samples. The next step involves ligation of adapters containing the sequencing recognition sites (A & B) followed by a sample specific barcode (ligation was performed according to GS FLX Shotgun DNA library preparation quick guide). Once MID containing adapters are ligated, samples can be pooled into a single tube for MPS (see below).

Approach 2: for hereditary breast cancer (2 genes: *BRCA1*, *BRCA2* - 111 amplicons) and familial aorta aneurism (3 genes: *FBN1*, *TGFBR1*, *TGFBR2* - 110 amplicons) two consecutive rounds of PCR were applied. In this approach adapter ligation is replaced by a second PCR step.

During the first PCR, gene-specific amplicons are generated, using primers modified at their 5′ end with a universal M13 linker sequence. In the first experiments (2 out of 10 experiments), we equimolarly pooled singleplex reactions. In further experiments the first amplification step was replaced by a multiplex PCR in which several amplicons of the same patient are combined (we typically aimed for 10-plex PCR reactions) to reduce the workload and consumable cost. After 1/1000 dilution of the PCR products, a second round of PCR is performed. In the second PCR, primers containing the common A or B sequence, a patient specific barcode sequence (MID) and a universal linker sequence (M13) were used to amplify the initial PCR products, thereby extending them with the sequences that are required to initiate sequencing and to distinguish reads from the different patients. Primer sequences, reaction conditions and constitution of the multiplex reactions are described by De Leeneer et al. [8] and Baetens et al. [9].

#### Pooling prior to sequencing

PCRs prior to pooling were performed in the presence of a saturating dye (LCgreen+, Idaho Technology Inc) on a real-time PCR instrument (CFX384, Bio-RAD). PCRs were normalized and equimolarly pooled in relation to the RFU data (endpoint fluorescence). This pool was purified on a High Pure PCR Cleanup Micro kit (Roche).

During optimization of the multiplex reactions FAM labeled MID primers were used to evaluate equimolarity between amplicons within one reaction and fluorescent peaks were separated on an ABI3730 capillary system.

### Sequencing reaction and data analysis

Emulsion PCR and sequencing reactions on the GS-FLX (454-Roche) were performed according to the manufacturer's instructions. On average 380,000 (range: 290,000–520,000) reads were obtained in a standard GS-FLX run and 1,000,000 when the Titanium chemistry was used (range: 800,000–1,200,000). In each experiment, a minimum of 90% of all reads mapped to the reference sequence. FASTA files were analyzed with the in house developed variant interpretation pipeline (VIP) software (version 1.3) [10].

Distribution plots and log-normal curve fitting were performed using the GraphPad PRISM 5 software. Statistical analysis of the potential bias introduced during emulsion PCR and pyrosequencing was performed using the R package. The mean of both relative coverages (obtained after sequencing on the GS-FLX) and relative fluorescent signals (obtained on capillary electrophoresis on an ABI3730) was used to center both data sets for each multiplex prior to principal component analysis to remove the effect of the different multiplex sizes.

## Results

### Calculation of coverage depth in function of sensitivity

With Sanger sequencing a two-fold (forward and reverse) coverage is considered to be sufficient for molecular diagnostics, provided that sequences are of high quality. At this moment there is no clear consensus on the required minimum coverage (MC) to reliably detect heterozygous variations using MPS technologies. Current guidelines typically suggest a 20-fold coverage [11], with little justification on the proposed value or how it would require adjustment depending on sequencing and analysis procedures or context. Because MPS is based on the sequencing of single, clonally amplified molecules, sampling effects need to be taken into account at low coverage. At one fold coverage there is a 50% chance to detect a heterozygous variant and a 50% (1/2^∧1^) chance to miss it. At two fold coverage, there is a 25% chance to detect only the mutant allele, 50% chance to detect both and 25% (1/2^∧2^) to detect only the wild type allele. Even at 10-fold coverage there is a chance of about 1/1000 (1/2^∧10^) to miss the variant allele completely. Since data analysis usually involves filtering out low frequency variants to reduce false positives resulting from sequencing errors (see below), the minimal number of reads for detection of heterozygous variants depends on the applied filter settings.

Table 1 shows an overview of the theoretically required minimal coverage (MC) to reliably detect heterozygous variants at varying minimum allele frequencies with a given power. Calculations were based on the following: the interpretation of a specific base has only two possible outcomes (equal to or different from the reference sequence). Theoretically, the probability to observe a variant in a specific number of reads (#Rv) out of all reads for a SAC (total coverage) can be derived from a binominal distribution with success probability equal to the expected mutant variant frequency in the total number of reads (50% for heterozygous variants without variant related alignment errors). The binomial distribution can also be used to tabulate the cumulative probabilities in function of the total coverage and the relative variant frequency that is deemed sufficient to indicate a real variant, i.e. above the filter level below which variants are thought to be sequencing errors (#Rv/total coverage). Hence, one can simply look up the coverage that is required for detecting a heterozygous variant at a minimum defined variant frequency with a predefined power. This coverage is referred to as the minimum coverage (MC) for a given SAC. To facilitate interpretation, power values (P) were converted into scores (Q) (similar to calculation of PHRED scores [12]): Q = −10*log(1−P).

**Table 1:** Overview of the required coverage to detect heterozygous variants, in function of the desired power (rows) and the level of filtering being applied (columns).

		sequencing error filter level						
Power (Q)		5%	10%	15%	20%	25%	30%	35%
90.00%	(10)	4	4	4	7	7	12	24
95.00%	(13)	5	5	8	8	11	18	30
99.00%	(20)	7	7	11	17	19	35	54
99.50%	(23)	8	12	12	18	26	42	71
99.90%	(30)	10	14	18	27	38	61	110
99.95%	(33)	11	15	19	28	42	68	117
99.99%	(40)	14	18	28	34	54	83	148
99.995%	(43)	15	19	30	42	61	92	165
100.00%	(50)	17	25	36	51	70	109	194

Not surprisingly, MC values increase as the required power to detect heterozygous variants increases. There is also a strong dependency on the sequencing error filter level: if only variants present in 30% of the reads are considered as true variants, a 61-fold MC is required, while a coverage depth of only 27 is needed if the filter threshold is lowered to 20% (both for or P = 99.90%, corresponding to a Phred score of 30, required for standard molecular diagnostics).

When plotting obtained variant frequencies vs. coverage of unfiltered data, the largest deviations from the binomial distribution are observed at the lower allele frequencies. Because the majority of such data points are sequencing errors, especially related to homopolymers (see below), dispersion can best be evaluated at frequencies above 50%. Allele specific amplification biases during sample preparation or emulsion PCR are the most likely cause of any remaining dispersion. A stepwise analysis starting from unfiltered variant data in one experiment (9721 variants) to determine the dispersion is shown in Supporting information Tool S2. We calculated the overall fraction of heterozygous variants with a frequency deviating from the expected 50% ratio, and this was estimated to be 10%, after correcting for sequencing errors being interpreted as heterozygous variants.

### Number of samples per run in function of MC

Determination of the required minimum coverage is not sufficient to calculate the number of SAC that can be analyzed with a given number of reads because the coverage may differ between SAC. In an ideal experiment, all SAC have exactly the same coverage, matching the theoretically determined required MC. In practice, some SAC will display a lower coverage than others. Since these require at least the MC as well, other SAC will have a higher coverage than absolutely required wasting sequencing capacity. The correction factor to convert the minimum coverage into the required average coverage can be derived from an evaluation of the distribution of the coverage.

**Figure 1:**
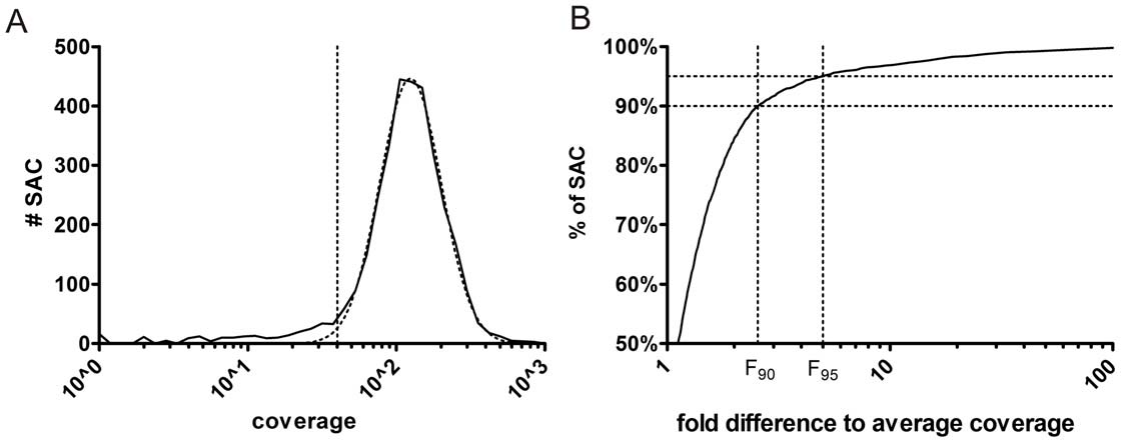
Coverage analysis. A) Distribution plot of the coverage observed in a pilot study representative for NGMD screening (full line) with 3300 sample amplicon combinations (SAC), derived from sequencing 30 patients for *FBN1*, *TGFBR1* and *TGFBR2*. The coverage across different SAC appears to be log normally distributed (R^2^ with best Gaussian fit (dashed line)>0.99). At low coverage (<40, vertical line), the distribution deviates from its Gaussian fit. This reflects a low number of reactions that failed to give a normal coverage. Analysis of these SAC may provide clues on how to further optimize the screening. B) Cumulative distribution plot of the relative coverage (expressed as a fold difference of each SAC to the average coverage). This plot allows determination of the correction factor by looking up the relative coverage for which the curve passes a given threshold, e.g. 90% for the calculation of F_90_.

Figure 1A plots the distribution of coverage to the number of SAC and shows that the variation in coverage depth is log normally distributed. Coverage data of 3300 SAC were used to generate this plot (3 genes: *FBN1*, *TGFBR1*, *TGFBR2* for 30 patients). Only at low coverage (<40), the distribution deviates from its Gaussian fit. This reflects a low number of reactions that failed to give a normal coverage. By calculating this variation in coverage depth, one can dictate how many extra reads are needed to cover all sequences at the required level. By plotting the cumulative distribution of the fold difference of the mean coverage to the SAC coverage, one can determine the correction factor by which the mean coverage needs to be multiplied in order to have a given fraction of SAC with at least the minimum coverage (Figure 1b). The value on the X-axis at which the histogram passes the 90% threshold is defined as the correction factor (F_90_). More stringent correction can be obtained by calculating a correction factor at higher thresholds (e.g. F_95_).

Supplemental Tool S2 provides an easy to use calculation template (MS Excel). Based on the coverage obtained in a proof of principle experiment, one can simply calculate the spread correction factor and the number of patients that can be screened in a run, ensuring sufficient power to detect heterozygous variants. The ‘spread correction factor sheet’ calculates the spread correction factor obtained (see also figure 1B) based on the coverage of different SAC in an experiment. In the ‘Samples per screening sheet’, additional requirements like predefined power, threshold for sequencing error filtering, instrument specifications and number of amplicons can be filled in. For example, for a *BRCA1/BRCA2* screening of 111 amplicons using P = 99.90%, threshold = 25% and spread correction factor 2.5 the tool determines that 83 samples can be screened in a single GS-FLX (Titanium chemistry) run with 90% of sequences covered sufficiently to provide a minimum power of 99.9%. This number decreases to 65 samples if 95% of the sequences need to be covered sufficiently.

### Emulsion PCR

**Figure 2:**
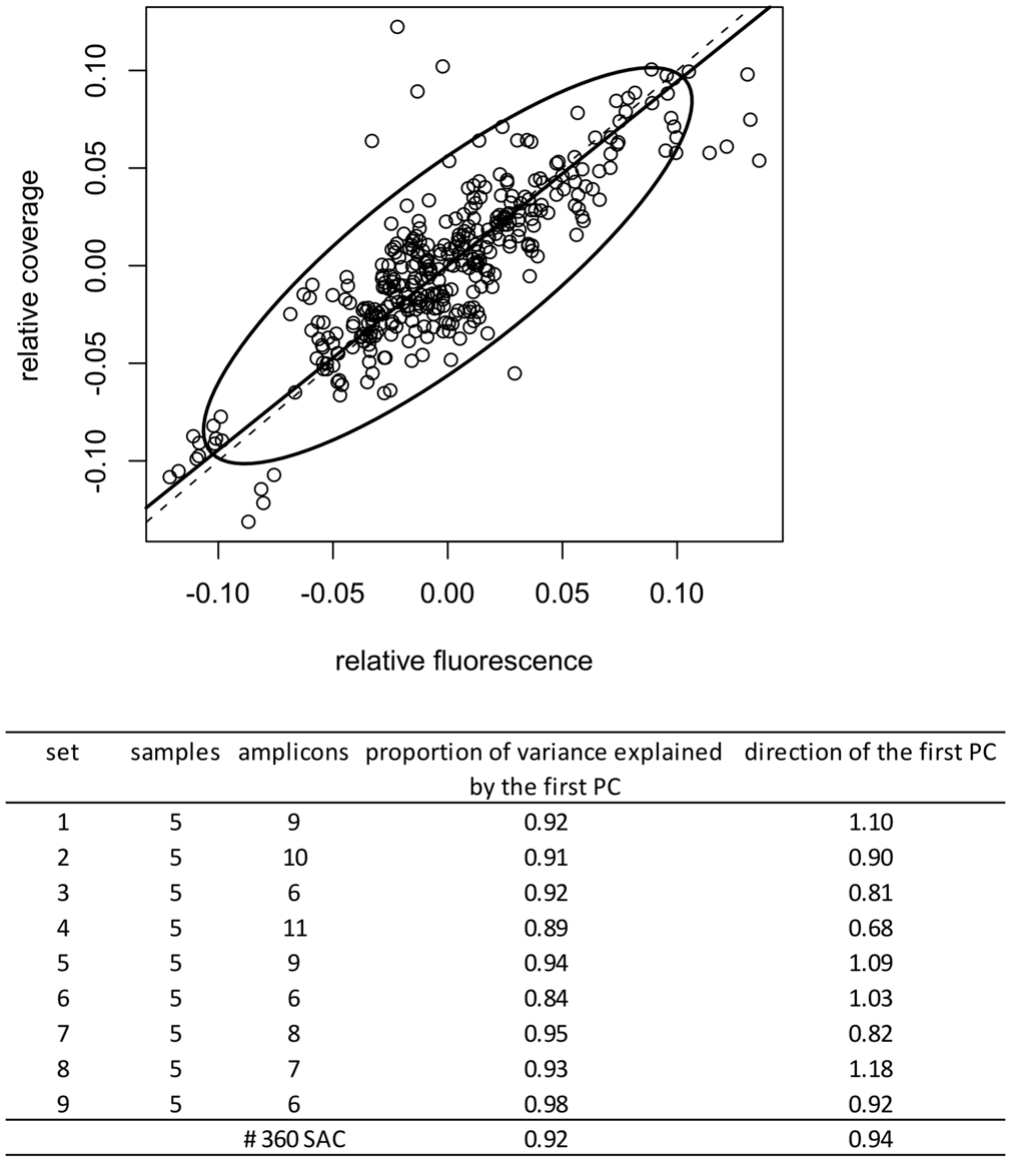
emulsion PCR and sequencing bias. Nine different fluorescently labeled multiplex PCRs (6 to 11-plexes), amplified on 5 different samples, were analyzed on a capillary sequencer to determine relative amplicon abundances prior to emulsion PCR and sequencing on a GS-FLX. Relative fluorescent signals were compared to their corresponding coverage values. The top panel shows the relative coverage in function of the relative fluorescence for the 360 SACs. The ellipse represents the 95% confidence region according to the multivariate normal distribution. The continuous line is the first principal component (PC) which indicates the direction of the largest variance in the sample: 92% of the variance of the sample can be explained by the first PC. The first PC lays very close to the first bisectrice (dashed line). Hence, there is a good 1∶1 relationship between the relative fluorescence and the relative coverage, indicating that a certain increase in relative fluorescence on average induces an equal increase in relative coverage. The table at the bottom summarizes results across all 9 multiplex PCRs (360 SACs). It shows that the first PC explains a large proportion of the variance of each multiplex (84%–98%): the majority of variation in coverage results from variations in input amounts (as determined by fragment analysis on a capillary sequencer).

We assumed a more narrow spread in coverage would be obtained by sequencing an equimolar pool of fragments or amplicons. To test the assumption that the emulsion PCR does not introduce a substantial bias we compared the relative peak intensities (determined by fragment analysis on ABI3730xl) of 9 different fluorescently labeled multiplex PCRs (6 to 11-plexes), amplified on 5 different samples (total of 360 SACs) with the corresponding relative coverage after sequencing. Overall there seems to be good 1∶1 relationship between the relative fluorescence and the relative coverage, indicating that a certain increase in relative fluorescence on average induces an equal increase in relative coverage (Figure 2). In contrast to the findings obtained for shotgun sequencing [13], our data indicate that sequencing bias is limited and that sequencing cost efficacy can be improved by generating more equimolar input pools.

**Figure 3:**
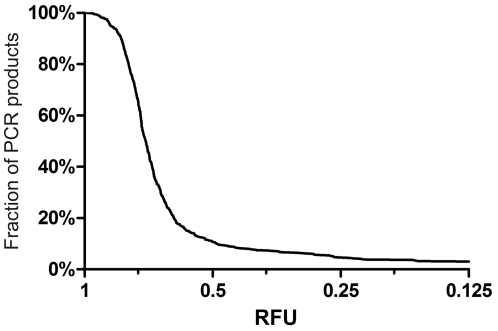
Analysis of amplicon abundance. This graph represents the distribution of the relative end point fluorescence intensities (RFU, relative to the maximum fluorescence), across 627 different qPCR reactions on a single sample. About 90% of reactions have RFU values of at least 0.5. This implies that if equal volumes of all PCR reactions are pooled, the concentration of 90% of amplicons will vary less than 2-fold. This fraction of amplicons can be increased to 96% by using a double volume for the PCRs in the 0.5–0.25 RFU range, and to 97% by using a quadruple volume for the PCRs in the 0.25–0.125 RFU range. The concentration of the remaining 3% of PCR reactions is too low to be efficiently used.

Equimolarity can be achieved by optimizing amplification conditions or by normalizing PCR product concentrations. Although normalization can potentially increase sequencing efficiency, one may lose on overall processing efficiency due to the required effort to normalize the SAC. With good primer design tools one should be able to get similar DNA quantities (as measured by end point fluorescence in a qPCR reaction with saturating DNA binding dye) for the 90% best assays. For such screenings, the majority of amplicons do not require any normalization and a significant portion of all remaining amplicons can be made equimolar by a simple normalization. Figure 3 shows the distribution of the relative end point fluorescence intensities (RFU, relative to the maximum fluorescence), across 627 different qPCR reactions on a single sample amplified for 15 genes associated with hearing loss. It is important to notice that comparison of end point fluorescence values is only valid for singleplex PCR products of comparable length.

### Sequence quality analysis

**Figure 4:**
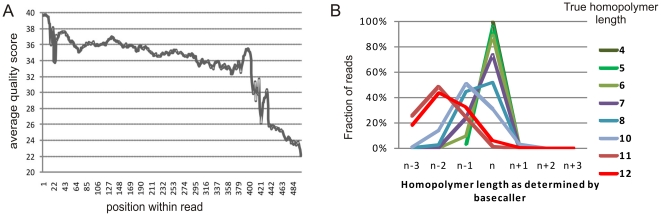
GS-FLX sequence quality analysis. a) Average quality score in function of the position within the reads for a representative dataset (full Titanium run with amplicons for breast cancer and for familial aorta aneurysmata screenings). Across the first 400 bp there is an average quality of 35.3 corresponding to a predicted error rate of 0.029%. b) Comparison of the observed homopolymer length in a series of sequencing runs to the expected length based on the reference sequence. Results are plotted as the fraction of reads having correct homopolymer length estimation (n), an underestimation of the homopolymer length (n−1, n−2, n−3) or an overestimation (n+1, n+2, n+3). The vast majority of reads for homopolymers of up to 6 repeats has correct length estimation, less than 2% are overcalls and less than 10% are undercalls. For homopolymers of 7 repeats, three quarters of the reads are correctly called and over 20% of the reads are interpreted to be missing one repeat. Only by filtering for low allele frequencies can these repeats be analyzed. At 8 repeats only about half of the reads are correctly called, at even larger homopolymer lengths only a minority of reads have a correct basecalling.

Sequence quality was determined using the GS-FLX basecaller. Quality scores per base were averaged across all reads within a single run (∼700,000 reads of 1 GS-FLX Titanium experiment for *BRCA1/2* and *FBN1*, *TGFBR1*, *TGFBR2* amplicons), and plotted in function of the sequenced base (Figure 4A). Because of the setup of this amplicon sequencing run, the number of reads longer than 400 bp was too low to provide accurate quality estimations in that range. Quality scores (Q) were converted into probabilities of erroneous basecalls (P) as follows: P = 10∧(−Q/10), corresponding to the better known Phred scores.

Pyrosequencing reactions are characterized by a low false call rate for substitutions, but also by a higher error rate for insertions and deletions – especially in homopolymeric regions [14]. A combination of quality and allele frequency filters may eliminate most errors, but fails to distinguish real insertions/deletions from sequencing errors in case of longer homopolymers (7 or more repeats) (Figure 4B).

## Discussion

As massively parallel sequencing has the ability to become the standard for next generation molecular diagnostics, more insight is urgently needed in the limitations of the technology and tools are required to standardize the quality of the diagnostic tests offered in various laboratories. In this study, we thoroughly evaluated data obtained with 10 GS-FLX experiments allowing us to shed light on a number of important issues and provide workarounds.

Current massively parallel sequencers offer a throughput per run that is insufficient for complete genome sequencing at affordable cost in a diagnostic setting, but mostly supersedes the requirements for targeted resequencing of single DNA samples. Strategies for next generation molecular diagnostics will therefore have to deal with both the selection of regions of interest and with sample multiplexing. Regions can be selected by either hybridization based enrichment or PCR amplification. Enrichment by capturing DNA fragments on oligonucleotides – on array (e.g. NimbleGen, Febit) or in solution (e.g. Agilent, Illumina) – has the advantage that many regions can be targeted in parallel (target multiplexing). While this allows enrichment of a high number of regions of interest (up to an entire human exome), it is well known to introduce large variations in coverage [15], [16]. In addition, enrichment is rarely complete: some regions are not captured whereas other unwanted regions may be copurified. The main drawbacks of this technology for molecular diagnostics are its high cost and the large quantities of high quality DNA that are required. Commercially available sample preparation approaches like Raindance, Fluidigm Acces array or more recently Haloplex PCR can increase throughput tremendously, but are less cost efficient for smaller experiments. Using the more classical approach of small scale, self-designed PCR assays has the advantage that the same set-up as for Sanger sequencing can be maintained, facilitating confirmation of the detected mutations afterwards. For these reasons, we evaluated the latter for NGMD.

Sample multiplexing can be achieved by physically separating samples in the sequencing reaction or by tagging the amplicons with different sample specific sequences during library preparation. Physical separation on current MPS instruments offers limited flexibility in the number of samples to be multiplexed (up to 16 in GS-FLX) and may reduce the available sequencing capacity by blocking parts of the available sequencing space. Therefore, a sample tagging approach is preferred. For applications where different samples are analyzed for different genes, no special multiplexing modifications need to be done when sequences can be easily attributed to the different samples based on correct alignment to the gene of interest. Four major amplification based approaches for NGMD are currently used worldwide: 1) PCR with fusion primers (GS-FLX), 2) PCR followed by adapter ligation (GS-FLX), 3) two consecutive rounds of PCR (GS-FLX), and 4) shearing of concatenated PCR products followed by adapter ligation (various MPS platforms). It must be noted that other approaches or variations on the methods described may be used as well. In this study, we evaluated approach 2 and 3. The main advantages of approach 2 are its simplicity and ease of set-up. The drawback is the large number of individual PCR reactions that need to be performed. Hence, we concluded that this approach is best suited if a screening only needs to be performed a few times or when results are quickly required and one cannot afford optimization. As soon as a few hundred samples need to be screened, approach 3 may be the preferred alternative. By multiplexing PCR reactions in approach 3, one can reduce the workload and consumable cost for sample preparation. Although optimization of multiplex PCR may be challenging, there is a good return in increased efficiency (in terms of cost and workload to prepare samples) for tests that will be run many times – as is the case in diagnostic sequencing. Further optimization may be achieved if the first and second round of PCR can be combined into a single PCR containing the two types of primers (inner target specific and outer sample specific primers).

Because of fundamental differences between the traditional and the so called next-generation sequencing methods, people are uncertain on how to deal with coverage and how to interpret variants, errors and quality scores. Despite the availability of some guidelines on required coverage provided by sequencing instrument suppliers, there was no theoretical framework to actually calculate the required minimum coverage. We here provide such a framework and implement it into a spreadsheet template that can be used to determine the required coverage and the number of patients that can be screened in a single run.

A number of sources of false positives and false negatives are identical for both Sanger and massively parallel sequencing and hence independent on the fold coverage. However, because MPS is based on the sequencing of single, clonally amplified molecules and uses a completely different sequencing chemistry, new types of error sources must be taken into account. Knowing the possible sources of error, one may optimize sample preparation and sequencing protocols, and take measures to adjust the data analysis pipeline for these new types of errors.

Table 1 shows an overview of the theoretically required MC to reliably detect heterozygous variants at varying minimum allele frequencies with a given power. Note that this theoretical MC value only accounts for allelic drop out due to sampling effects and that it should be treated as a lower limit for the actual MC that may be larger because of additional variation affecting allele frequencies. Because of inter-lab variation we cannot propose a single value for the required minimum coverage, but labs can determine their own MC value based on their sequencing error rate (filter setting) and the required power to detect variants (Table 1, Supplemental Tool S1). When new to NGMD, filtering at 25% and aiming for 99.9% power (resulting in an MC of 38) may be a good starting point. A 5-fold coverage is expected to be sufficient to tolerate occasional sequencing errors when screening for homozygous variations only.

Based on the strategies and methods described in this paper we successfully developed and validated the screening of the complete coding region of the *BRCA1* and *BRCA2* genes in a diagnostic setting [8], demonstrating the feasibility of performing more efficient molecular diagnostics using massively parallel sequencing. The application of massive parallel sequencing for clinical sequencing of *BRCA1/2* on the Illumina GAII has been recently described [17], [18]. We agree with Morgan et al., that the major remaining hurdle is the availability of data analysis tools that provide the required high quality for in-vitro diagnostics and that are really tailored towards a routine diagnostic setting. The availability of commercial software packages and the advent of smaller scaled MPS instruments such as the GS-Junior and Illumina Miseq and the development of the so-called third generation sequencers like Ion Torrent are expected to push this new sequencing technology into the field of diagnostics, starting with the multigenic disorders for which there are no good alternatives available at this moment. However, because of its proven track record, its superior flexibility and its large install base, Sanger sequencing is unlikely to be replaced in the near future for smaller screening projects and it will remain a valuable technology for confirmation of mutations observed by other technologies.

## Supporting Information

Tool S1 NGMD calculator.
(PDF)

Tool S2 Allele frequency analysis.
(XLSX)
