# Supplementary material for: Practical Tools to Implement Massive Parallel Pyrosequencing of PCR Products in Next Generation Molecular Diagnostics
Source: PLoS One. 2011 Sep 30;6(9):e25531. doi: 10.1371/journal.pone.0025531 (PMC3184136; doi:10.1371/journal.pone.0025531)
Supplement: Tool S1 — NGMD calculator. (PDF) [file pone.0025531.s001.pdf]

Allele frequency analysis

Dataset

- breast cancer
- familial aorta aneursim
- 1 full GS-FLX run
- 9721 variants

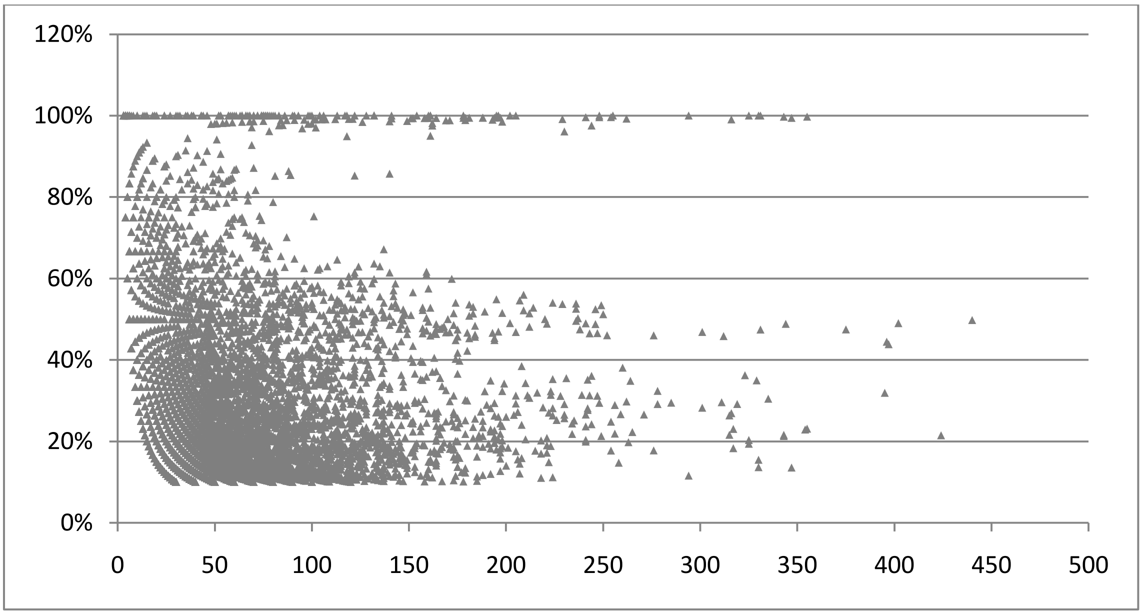

Filtering

Variation in allele frequencies for heterozygote variants is disturbed by sequencing errors that occur at elevated rates. The full dataset was trimmed for variants with

- quality < 30
- homopolymer length >= 6

After filtering for likely sequencing errors 3642 variants (37%) remain.

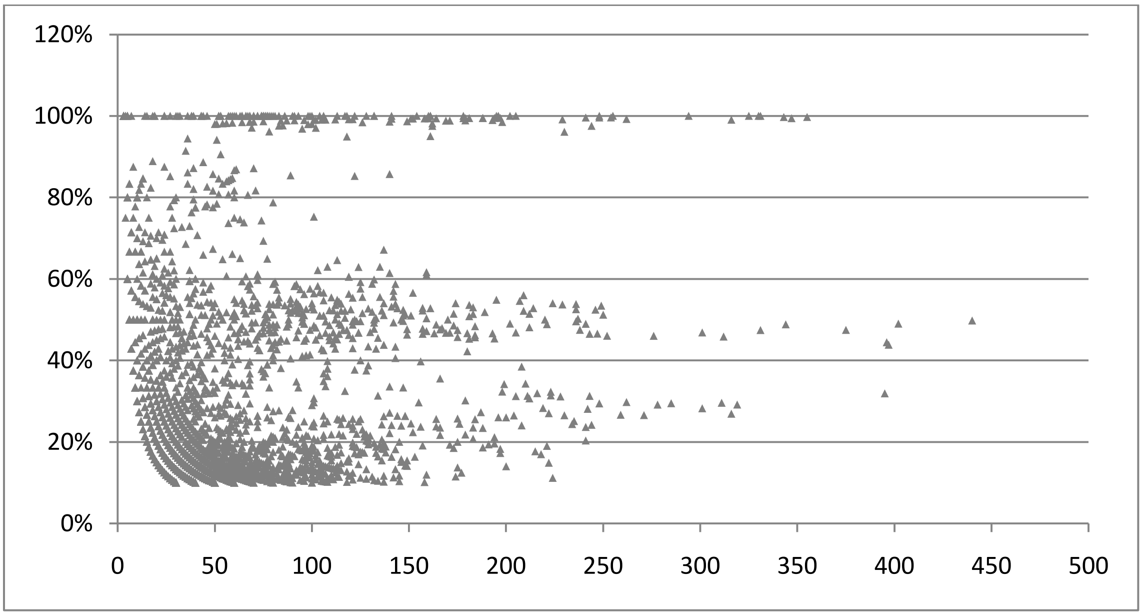

Allele frequency binning

To evaluate whether allele frequencies for heterozygote variants fluctuate randomly around the theoretical value of 50%, variants were binned into allele frequency ranges: <20%, [20-40%[, [40-60%],]60-95%], >95%. Variants that occurred in at least 5 samples were classified as having a systematic allelic bias if the number of samples with an allele frequency in the second (green) or fourth (red) bin was higher than the number of samples with that variant in the bin around 50%.

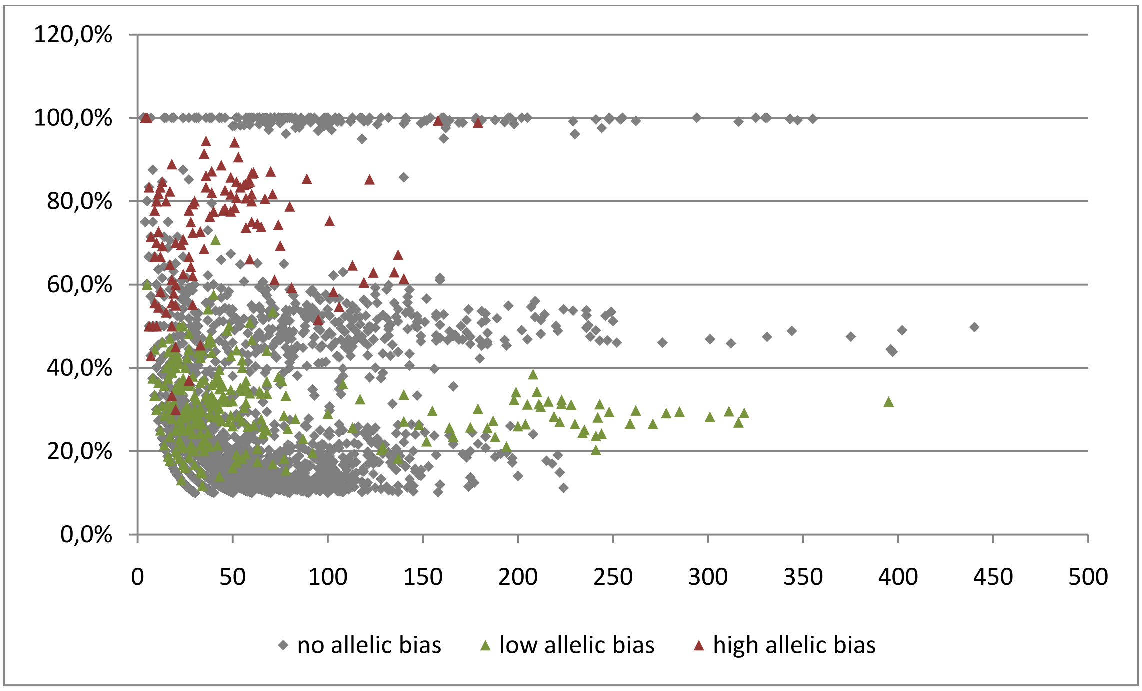

Out of 922 unique variants, 185 occurred in at least 5 samples. Of these, 13 (7.0%) and 6 (3.2%), respectively, showed a decreased (green) or increased (red) allele frequency. Because of sequencing errors in the lower allele frequency range, the occurrence of non-random allelic bias is expected to be closer to the occurrence rate of increased allele frequency than to that of decreased allele

frequency. Correcting for sequencing problems like those observed in the problematic amplicon BRCA2_11_19 (3 variants with skewed allele frequencies), the overall fraction of real heterozygous variants with allele frequencies deviating from the expected 50% ratio [40-60%] is estimated at 5%.

	<20	[20-40[	[40-60]	]60-95]	>95	occurrence
BRCA1_11_10 -262c	8%	58%	33%			12
BRCA1_18 -134-	13%	81%	6%			16
BRCA2_10_07 -22a	22%	67%	11%			9
BRCA2_11_19 -182a	11%	78%	11%			9
BRCA2_15 -9t	5%	74%	21%			19
BRCA2_26 -27t		86%	14%			14
FBN1_exon_16_2 -90a	18%	59%	18%	5%		22
FBN1_exon_23 -225a	3%	73%	25%			40
FBN1_exon_28_2 -59-----	25%	75%				8
FBN1_exon_38 -21t	15%	62%	23%			39
FBN1_exon_53 -54t	14%	66%	21%			29
TGFBR1_3_UTR_1 -27g		100%				41
TGFBR1_exon_7 -33t	6%	76%	18%			34
BRCA2_03 -23a			42%	50%	8%	12
BRCA2_11_19 -178-			31%	69%		13
BRCA2_11_19 -199-		22%	28%	44%	6%	18
BRCA2_16 -9t			21%	79%		19
FBN1_exon_18_1 -117c			28%	61%	11%	18
FBN1_exon_31_2 -172-				100%		41

Remaining variation

After exclusion of variants with demonstrated allele frequency bias and variants with allele frequencies below 20% (all of which were shown to be false positives, i.e. PCR and sequencing errors), 623 variants with a coverage of at least 20 (to allow for reliable allele frequency estimation) remained.

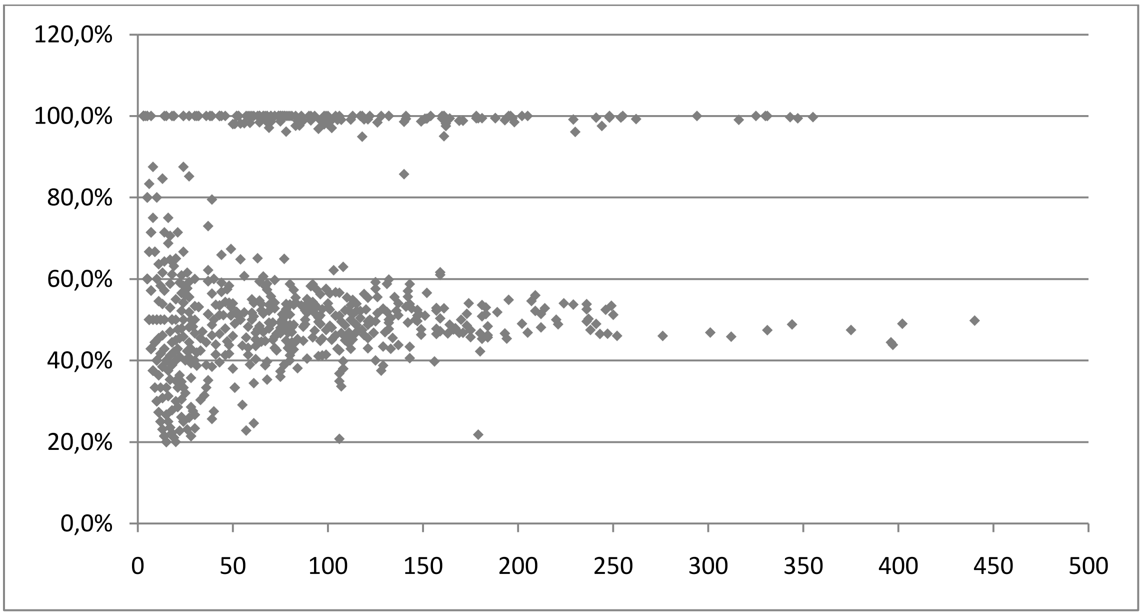

This dataset allowed for evaluation of residual allele frequency bias.

allele frequency	count	fraction
>95	147	24%
]60-95]	24	4%
[40-60]	384	62%
[20-40[	68	11%

Based on this data, and correcting for sequencing errors being interpreted as heterozygous variants, the overall fraction of heterozygous variants with allele frequencies deviating from the expected 50% ratio [40-60%] is estimated at 10%.
